# Meiosis Resumption of Immature Human Oocytes following Treatment with Calcium Ionophore *In Vitro*

**DOI:** 10.22074/cellj.2021.7130

**Published:** 2021-03-01

**Authors:** Elham Fazeli, Ahmad Hosseini, Mohammad-Hasan Heidari, Fattaneh Farifteh-Nobijari, Mohammad Salehi, Hojjat-Allah Abbaszadeh, Hamid Nazarian, Zahra Shams Mofarahe, Saman Ayoubi, Sara Hosseini, Mona Shayeghpour, Mojgan Bandehpour, Marefat Ghaffari Novin

**Affiliations:** 1. Department of Biology and Anatomical Sciences, School of Medicine, Shahid Beheshti University of Medical Sciences, Tehran, Iran; 2. Mehr Fertility Research Center, Guilan University of Medical Sciences, Rasht, Iran; 3. Cellular and Molecular Biology Research Center, Shahid Beheshti University of Medical Sciences, Tehran, Iran; 4. Genetics and In Vitro Assisted Reproductive (GIVAR) Center, Erfan Hospital, Tehran, Iran; 5. Department of Medical Biotechnology, School of Advanced Technologies in Medicine, Shahid Beheshti University of Medical Sciences, Tehran, Iran; 6. Infertility and Reproductive Health Research Centre, Sara Hospital, Tehran, Iran

**Keywords:** Calcium Ionophores, In Vitro Oocyte Maturation Techniques, Maturation-Promoting Factor, Meiotic Spindle, Mitogen-Activated Protein Kinase

## Abstract

**Objective:**

*In vitro* maturation (IVM) of human oocytes is used to induce meiosis progression in immature retrieved oocytes. Calcium (Ca2+) has a central role in oocyte physiology. Passage through meiosis phase to another phase is controlled by increasing intracellular Ca2+. Therefore, the current research was conducted to evaluate the role of calcium ionophore (CI) on human oocyte IVM.

**Materials and Methods:**

In this clinical trial study, immature human oocytes were obtained from 216 intracytoplasmic sperm injection (ICSI) cycles. After ovarian stimulation, germinal vesicle (GV) stage oocytes were collected and categorized into two groups: with and without 10 µM CI treatment. Next, oocyte nuclear maturation was assessed after 24–28 hours of culture. Real-time reverse transcription polymerase chain reaction (RT-PCR) was used to assess the transcript profile of several oocyte maturation-related genes (MAPK3, CCNB1, CDK1, and cyclin D1 [CCND1]) and apoptotic-related genes (BCL-2, BAX, and Caspase-3). Oocyte glutathione (GSH) and reactive oxygen species (ROS) levels were assessed using Cell Tracker Blue and 2’,7’-dichlorodihydrofluorescein diacetate (H2DCFDA) fluorescent dye staining. Oocyte spindle configuration and chromosome alignment were analysed by immunocytochemistry.

**Results:**

The metaphase II (MII) oocyte rate was higher in CI‐treated oocytes (73.53%) compared to the control (67.43%) group, but this difference was not statistically significant (P=0.13). The mRNA expression profile of oocyte maturation-related genes (MAPK3, CCNB1, CDK1, and CCND1) (P<0.05) and the anti-apoptotic BCL-2 gene was remarkably up-regulated after treatment with CI (P=0.001). The pro-apoptotic BAX and Caspase-3 relative expression levels did not change significantly. The CI‐treated oocyte cytoplasm had significantly higher GSH and lower ROS (P<0.05). There was no statistically significant difference in meiotic spindle assembly and chromosome alignment between CI treatment and the control group oocytes.

**Conclusion:**

The finding of the current study supports the role of CI in meiosis resumption of human oocytes. (Registration Number: IRCT20140707018381N4)

## Introduction

*In vitro *maturation (IVM) of human oocytes is a valuable technique in assisted reproductive technology (ART). During the IVM procedure, immature oocytes are retrieved from small antral follicles and then meiosis progression occurs in the laboratory (1).

IVM could be an appropriate alternative in various conditions such as patients who have poor ovarian response to gonadotropin stimulation, high numbers of antral follicles, polycystic ovarian syndrome, egg factor problems with only germinal vesicle (GV) oocytes in their stimulation cycles, and those who suffer from cancer who intend to cryopreserve their oocytes prior to the onset of cancer treatment (2). However, the developmental potential of oocytes reduces after IVM compared to *in vivo* matured oocytes (2, 3).

The IVM process differs from natural ovulation, in which an oocyte resumes meiosis after the luteinizing hormone (LH) surge. Therefore, IVM changes the usual timeline of cytoplasmic and nuclear maturation processes (2). Many events occur during oocyte maturation, such as cytoplasmic reorganization, cytoskeletal dynamics, and meiotic resumption, which are essential for regular fertilization and embryonic development (1). The preovulatory LH surge by the activation of a signaling cascade leads to meiosis resumption in the oocyte *in vivo* (4). 

Oocyte meiotic progression involves protein phosphorylation pathways that are regulated via cyclindependent kinases (CDKs) (5). Maturation-promoting factor (MPF) consists of two subunits, CDK1 and cyclin B, and is a key factor in meiotic resumption (6). MPF activity depends on the availability of cyclin B and phosphorylation status of CDK1 (7). Mitogen-activated protein kinase (MAPK) signalling is involved in the oocyte maturation process (5). In human oocytes, MAPK is inactive in the GV stage oocytes, reaches its highest activity in the metaphase II (MII) stage, and has reduced activity after pronucleus formation (8). MAPK signalling regulates MPF activation, and the MAPK inhibitors block germinal vesicle breakdown (GVBD) in oocytes (6). High cyclic adenosine monophosphate (cAMP) in oocytes promote meiotic arrest until ovulation (7). In the somatic follicular cells, cAMP synthesis is catalysed from ATP by the adenylyl cyclase enzyme and is transferred into the oocyte via gap junctions (5). During oocyte meiotic arrest, high levels of protein kinase A (PKA), a cAMP cell cycle mediator, inhibit the CDK1 subunit of MPF (5, 7). In the *in vivo* study, a preovulatory LH surge starts the meiosis resumption by destructing oocyte-somatic cell communication, preventing cAMP transfer, and it also activates the MAPK cascade in cumulus cells (5). The MAPK signalling cascade, which is known as extracellular signal-regulated kinases (ERK1/2) elevates phosphodiesterase, a cAMP degrading-endzyme activity in the oocyte, and thereby decreases cAMP in the oocyte. cAMP-degrading in the oocyte reduces PKA activity (7). A decrease in PKA activity leads to dephosphorylation of the inhibitory sites of CDK1 and results in MPF activation. Activated MPF phosphorylates histones, lamins, and other cellular components. Subsequently, meiosis is resumed (9).

Glutathione (γ-glutamyl-cysteinyl glycine, GSH) is an intracellular free thiol, which is an essential nonenzymatic antioxidant within cells. GSH levels in the oocyte are an excellent cytoplasmic maturation marker after IVM (10). A critical role of GSH, as an antioxidant, is defending the oocyte against oxidative injuries through decreasing reactive oxygen species (ROS) production in mitochondrial metabolism (11). The oxidative damage status in oocytes is one of the essential markers to assess the health of the oocyte. ROS damage in the oocyte can lead to unexpected apoptosis and subsequent arrest in embryonic development (12).

Reduction in anti-apoptotic factors, such as BCL-2, leads to an elevation in pro-apoptotic factors, including BAX. These apoptotic factors cause oocyte apoptosis (13). The BAX/BCL-2 ratio regulates a cascade of molecular events that determine the cell’s fate (survival or apoptosis). Increased BAX/BCL-2 alters mitochondrial membrane polarization and results in cytochrome C influx from mitochondria into the cytosol, which involves inactivation of the initiator (8 and 9) and effector (3, 6, and 7) caspases in oocytes (14). The BCL-2 protein kinase sites are phosphorylated during the G2 to M transition of the cell cycle. Phosphorylation of Thr-56, Thr-74, and Ser-87 BCL-2 residues inhibit proteasome function and prevent apoptosis. It has been suggested that MAPK and MPF have an essential role in this process (15).

Calcium ionophore (CI) is a fat-soluble molecule that increases cytosolic calcium (Ca^2+^) by transferring Ca^2+^ from the plasma membrane to the cytoplasm (16). Ca^2+^ signaling is a key factor in the physiology of oocytes from oogenesis to maturation and fertilization. The passage through the meiosis phase to another phase is controlled by cell checkpoints, which act in many species by increasing intracellular Ca^2+ ^levels (17). During fertilization, sperm-induced elevation in intracellular Ca^2+^ is necessary for oocyte activation, which is a trigger for transforming an oocyte into an embryo (1). Also, Ca^2+^ changes the activity of specific transcription factors in the nucleus, and these factors affect chromatin structure and, as a result, gene expression (18).

Previous studies have shown the relationship between Ca^2+^ and GVBD. Increasing Ca^2+^ during GVBD can indicate a correlation between intracellular Ca^2+^ and oocyte maturation in different species of mammals (17). Furthermore, it is reported that the duration of Ca^2+^ oscillation increases during oocyte maturation. Oocytes with increases levels of cytosolic Ca^2+^ have higher spontaneous parthenogenetic activation (19). 

IVM conditions may influence the oocyte’s developmental competence. In general, there is no accepted procedure in infertility clinics for the IVM of oocytes. On the other hand, IVM, as a clinical approach, should be optimized for the future (2). The current study was carried out to clarify the role of CI on IVM of human oocytes.

## Materials and Methods

Ethics approval for the current randomized clinical trial study was given by the Ethics Committee at Shahid Beheshti Medical University, Tehran, Iran (IR.SBMU. MSP.REC.1396.416). Participants gave verbal and written consent for study participation. All procedures in this research were in accordance with the ethical guidelines of responsible institutional and national committees that involve human experimentation (IRCT20140707018381N4).

### Patients

The oocytes were donated for the current study by the patients of the Genetics and In Vitro Assisted Reproductive (GIVAR) Center at Erfan and Taleghani Hospitals (Tehran, Iran) between October, 2017-November, 2018.

A total of 552 GV oocytes from 216 intracytoplasmic sperm injection (ICSI) procedures were included in the current study. These oocytes were not suitable for the ICSI procedure. All women participants were ≤40 years of age (mean: 32.13 ± 4.96 years). Cycles diagnosed as male factor infertility (n=135), tubal factor infertility (n=70), uterine factor (n=4), and unexplained infertility (n=7) were included in the current study. Women who suffered from polycystic ovarian syndrome, endometriosis, and genetic disorders were excluded from this study.

### Ovarian stimulation protocol and oocyte retrieval

Ovarian stimulation was carried out using the long protocol. Briefly, gonadotropin-releasing hormone (GnRH) agonist (Superfact, Aventis Pharma, Germany) was adminstered on day 21 of the menstrual cycle. rFSH (Gonal-F, Merck Serono, Germany) was injected subcutaneously each day (150–300 IU/day) after the third day of menstrual bleeding for a duration of five days. 

For triggering ovulation, intramuscular administration of 10000 IU units of human chorionic gonadotropin (hCG) (Ovitrelle, Merck Serono Europe; Pregnyl, Organon) was performed when one of the follicles reached >18 mm in size as viewed by ultrasound. Transvaginal oocyte pickup via ultrasound guidance was carried out 36–38 hours following the hCG injection.

After oocyte retrieval, the oocytes were denuded by brief exposure to hyaluronidase (LifeGlobal) and frequent pipetting. Then, oocytes were evaluated under an inverted microscope for nuclear maturation assessment: i. GV stage showed a germinal vesicle in the cytoplasm, ii. meiosis I (MI) stage did not show any germinal vesicle in the ooplasm and first polar body (PB) in the perivitelline space, and iii. MII stage showed the presence of the first PB in the perivitelline space.

### *In vitro *maturation 

A total of 552 GV stage oocytes were obtained from women who had an adequate number of MII oocytes after oocyte retrieval (>80%). Dimethyl sulphoxide (DMSO) was used to dissolve the CI A23187 (Sigma Aldrich; St. Louis, MO, USA) according to the manufacturer’s protocol. Just before IVM, individual oocytes were transferred to 50 μL droplets that contained 10 µM CI of a stock solution diluted in culture medium (Global R, Life Global) for 15 minutes based on an artificial oocyte activation protocol (20). Then, the oocytes were washed in two, 50 μL droplets of culture medium. In the control group, GV oocytes were not exposed to CI. Oocytes from the treated and control groups were transferred to 50 μL droplets of culture medium (Global R, LifeGlobal) under mineral oil (LifeGlobal) and incubated in 6% CO_2_ air atmosphere at 37˚C. After 24-28 hours, oocyte maturation was assessed. Oocytes with the first PB (MII stage) were used for this study. 

### RNA isolation and cDNA synthesis

*MAPK3, CDK1, CCNB1, cyclin D1 (CCND1), BCL2, BAX, Caspase-3*, and* β-actin* gene expressions were assessed using real-time reverse transcription polymerase chain reaction (RT-PCR) in the IVM oocytes at the MII stage. Reverse transcriptions of samples were carried out as explained previously (21). In summary, a total of 78 oocytes (39 oocytes in each group) were washed in phosphate-buffered saline (PBS, Invitrogen Corp.) + 1% polyvinyl alcohol (PVA), and pooled into six Eppendorf tubes (13 oocytes in each microtube) with 1.5 µL of lysis buffer to isolate the RNA from the oocytes. The Eppendorf tubes were stored at -80˚C. Next, we added 5 μL nucleasefree water and 3 µL random hexamer to the Eppendorf tubes and placed them in a Bio-Rad thermocycler.

Complementary DNA (cDNA) synthesis was performed with 10 mmol/L dNTP, 200 U RT enzyme, 10 U RNase inhibitor, and 5× RT buffer in a total reaction volume of 21 µL for 10 minutes at 25˚C, 15 minutes at 37˚C, 45 minutes at 42˚C, and 10 minutes at 72˚C followed by overnight incubation at 4˚C. 

The investigated genes (*MAPK3, CDK1, CCNB1, CCND1, BAX, BCL-2*, and *Caspase-3*) and the internal control (*β-actin*) were amplified as follows. We added 1 μg cDNA, 3 μL nuclease-free water, 5 μL Master Mix (Amplicon, Denmark), and 10 nmol specific forward and reverse primers (Table 1) to the PCR Eppendorf tubes and processed them for 5 minutes at 94˚C, 30 seconds at 94˚C, 30 seconds at 60˚C, and 45 seconds at 72˚C and 40 extension cycles. The amount of RNA was visualized after loading the samples. The amplification products were visualized on agarose gel electrophoresis under short UV.

### Real-time RT-PCR analysis 

In order to quantify *MAPK3, CDK1, CCNB1, CCND1, BAX, BCL-2*, and *Caspase-3* gene expressions, realtime RT-PCR was performed in 13 μL of reaction buffer that contained synthesized cDNA, forward and reverse specific primers (1 mmol/L for each gene), and DNA Master SYBR Green I mix. The gene amplification program included 2 minutes at 95˚C, 5 seconds at 95˚C, 30 seconds at 60˚C, 10 seconds at 72˚C, and 40 extension cycles. The experiment for each sample was carried out in three replicates. Relative Expression Software Tool (REST, version 2009) was applied to calculate the expression of each of the investigated genes.

### Glutathione and oxidative stress 

The IVM-MII oocytes were collected from each group to determine their intracellular GSH (20 oocytes in each group) and ROS (23 oocytes in each group) levels by previously described methods (22). Briefly, the GSH and ROS content of the oocytes were detected using Cell Tracker Blue (CMF2HC; 4-chloromethyl-6, 8-difluoro7-hydroxycoumarin; Invitrogen), and H2DCFDA (2’,7’-dichlorodihydrofluorescein diacetate; Invitrogen) fluorescent dyes. Oocytes were transferred to a 30 µL PBS droplet that consisted of 10 µM Cell Tracker Blue, 10 µM H2DCFDA, and 1 mg/mL PVA in the dark at 37˚C for 45 minutes followed by three washes in PBS + 1% PVA. The samples’ intracellular GSH and ROS concentrations were observed as blue and green fluorescence under a fluorescence microscope (Labomed Lx 400; Labo America). The GSH and ROS contents were detected by 370 nm and 460 nm ultraviolet filters, respectively. Fluorescence images of oocytes were recorded as TIFF format graphics files and evaluated by ImageJ software (NIH, Bethesda, MD, USA), version 1.41.

### Immunocytochemistry

Immunocytologic staining of the spindle structure and chromosome arrangement in the IVM-MII oocytes (10 oocytes in each group) was carried out using a previously described method (23). Briefly, MII stage oocytes were treated for about 30 seconds by Tyrode’s acidic solution (pH=2.5) at room temperature to remove the zona pellucida. Next, 4% paraformaldehyde in PBS (pH=7.4) was applied for 30 minutes at 4˚C to fix the oocytes. Following three washes in PBS + 0.02% Tween 20, oocyte membrane permeabilization was induced by 0.25% Triton X-100 for 60 minutes at room temperature. Then, the oocytes were exposed to 4N HCl for 30 minutes at room temperature, followed by 0.1 M Tris-HCl for neutralization. The oocytes were transferred to a blocking solution that contained 2% bovine serum albumin (BSA, Sigma Aldrich; St. Louis, MO, USA) + 0.02% Tween 20 in PBS for 60 minutes at room temperature. Subsequently, the oocytes were placed in mouse monoclonal anti-β-tubulin antibody (1/100 dilution, Sigma Aldrich; St. Louis, MO, USA) in the blocking solution overnight in a humidified chamber at 4˚C. After several washes, meiotic spindle staining was carried out following 30 minutes incubation at room temperature of the oocytes with conjugated goat anti-mouse (IgG) fluorescein isothiocyanate (FITC) at 1/100 dilution (Sigma Aldrich; St. Louis, MO, USA) in the dark. After several washes, the oocytes were placed in 10 mg of propidium iodide (PI; Sigma Aldrich; St. Louis, MO, USA) for chromatin staining for 20 minutes. The samples were individually mounted on microscope slides and a coverslip and etched rings were applied to prevent the samples from being ruptured by the coverslip. The slide was observed under a fluorescent microscope (Labomed Lx 400; Labo America) and the chromosomes, and spindle configurations were defined as normal (aligned chromosomes at the metaphase plate with barrelshaped spindles) or abnormal (misaligned chromosomes in the metaphase plate with non-barrel-shaped spindles).

### Statistical analysis

The t test and chi-square test using SPSS (SPSS, Chicago, IL, USA) software (version 16.0) was applied to analyse differences between the two groups. Mean ± standard deviation (SD) and percentages were used to the express data. A p-value <0.05 was considered statistically significant.

**Table 1:** Primer sequences used in real-time RT-PCR


Gene	Sequence (5´-3´)	Length	GC%	Tm (˚C)

*MAPK3*	F: ATTGCCGATCCTGAGCATGACCAC	24	54.2	65
*MAPK3*	R: CAGATGTCGATGGACTTGGTATAG	24	45.8	58
*CDK1*	F: GGATGTGCTTATGCAGGATTCC	22	50.00	59.44
*CDK1*	R: CATGTACTGACCAGGAGGGATAG	23	52.17	59.42
*CCNB1*	F: GAAGATCAACATGGCAGGCG	20	55.00	59.62
*CCNB1*	R: GCATTTTGGCCTGCAGTTGT	20	50.00	60.25
*CCND1*	F:CATGCGGAAGATCGTCGCCACC	22	63.6	66
*CCND1*	R: CTCCTCCTCGCACTTCTGTTCC	22	59	61.5
*BAX*	F: GGAGGAAGTCCAATGTCCAG	20	55	59.505
*BAX*	R: GGGTTGTCGCCCTTTTCTAC	20	55	60.856
*BCL-2*	F: GCTATAACTGGAGAGTGCTGAAG	23	47.8	57.7
*BCL-2*	R: CATCACTATCTCCCGGTTATCGT	23	47.8	58.5
*Caspase-3*	F: GACATCTCGGTCTGGTACAGATGTGC	26	53.9	63.5
*Caspase-3*	R: ‘TTCACCATGGCTCAGAAGCACAC	23	52.2	62.5
*β-actin *	F: AGAGCTACGAGCTGCCTGAC	20	60	64
*β-actin *	R: AGCACTGTGTTGGCGTACAG	20	55	62


RT-PCR; Reverse transcription polymerase chain reaction, GC; Guanine-cytosine, and Tm; Melting temperature

## Results

### Effects of pre-*in vitro *maturation CI treatment on percentage of *in vitro*-matured human oocytes after 24–28 hours

Overall, 216 couples participated in this study (Table 2). Out of 552 GV oocytes, 390 (70.65%) reached the MII stage and 96 (17.39%) arrested in the MI stage. There were 50 (9.05%) oocytes that arrested in the GV stage and 16 (2.89%) oocytes were degenerated. Although the MII oocyte rate was higher in CI‐ treated oocytes (73.53%) compared to the control group (67.43%), this difference was not statistically significant (P=0.13). The GV arrested oocyte rate (CI‐ treated oocytes: 8.24% and control: 9.96%, P=0.06), oocyte degeneration rates (CI‐treated oocytes: 2.40% and control: 3.44%, P=0.46), and arrested MI oocyte rates (CI‐treated oocytes: 15.80% and control: 19.15%, P=0.87) after IVM was not statistically significant between CI‐treated oocytes and the control group (Table 3). This finding suggested that CI treatment significantly affected the first PB extrusion in human oocytes. 

**Table 2:** Baseline characteristics of the study population


Variables	Group	
	Control	CI-treated
	n(%) or mean ± SD (range)	n(%) or mean ± SD (range)

Number of cycles	112	104
Female age (Y)	32.63 ± 4.84 (20–40)	31.61 ± 5.07 (21-40)
Cause of infertility		
Male factor	68 (60.71)	67 (64.42)
Tubal factor	38 (33.92)	32 (30.76)
Uterine factor	1 (0.89)	3 (2.88)
Unexplained	5 (4.46)	2 (1.92)
Number of total retrieved oocytes	12.17 ± 6.85 (2–29)	13.06 ± 7.10 (1-29)
GV retrieved oocytes	2.13 ± 1.38 (1–7)	2.67 ± 2.13 (1-12)
Degenerated retrieved oocytes	0.64 ± 1.31 (0-6)	0.62 ± 1.04 (0-6)
MI retrieved oocytes	0.90 ± 1.36 (0-6)	1.29 ± 1.66 (0-6)
MII retrieved oocytes	8.83 ± 6.28 (0-28)	9.42 ± 5.98 (0-27)


The t test was applied for statistical analysis. There was no statistically significant difference in any parameter between the CI-treated and control groups. CI; Calcium ionophore, Ns; Not significant, GV; Germinal vesicle, MI; Metaphase I, MII; Metaphase II, and SD; Standard deviation.

**Table 3:** Meiotic maturation of human oocytes after 24-28 hours of culture


Group	No. of GV cultured	GV arrest	Degenerated	MI	MII
	N (mean ± SD)	N (mean ± SD)	N (mean ± SD)	N (mean ± SD)	N (mean ± SD)

CI-treated oocytes	291 (2.82 ± 2.34)	24 (0.23 ± 0.52)	7 (0.06 ± 0.32)	46 (0.44 ± 0.76)	214 (2.07 ± 1.71)
Control	261 (2.33 ± 1.52)	26 (0.23 ± 0.46)	9 (0.08 ± 0.35)	50 (0.44 ± 0.88)	176 (1.57 ± 1.27)


The t test was applied for statistical analysis. There was no significant difference in the meiotic maturation rate between the two groups. CI; Calcium ionophore, GV; Germinal vesicle, MI; Metaphase I, MII; Metaphase II, and SD; Standard deviation.

### Effects of pre *in vitro *maturation calcium ionophore treatment on nuclear maturation and apoptosisrelated gene expression levels of in vitro-matured human oocytes

**Fig.1:**
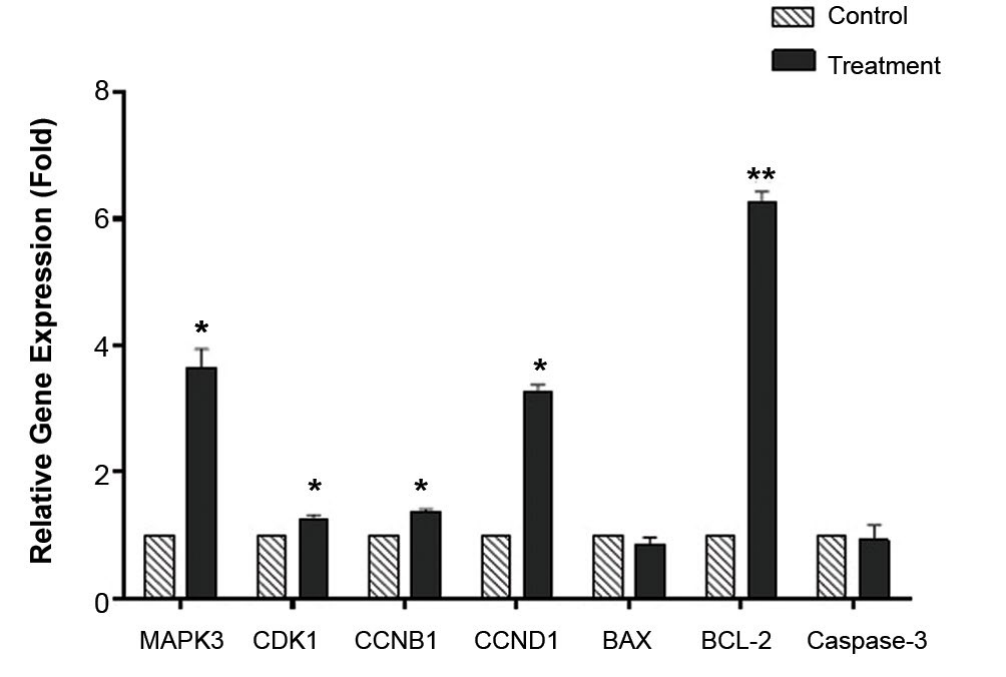
Nuclear maturation and apoptosis-related gene mRNA expressions of human oocytes. The relative expression levels of mitogen-activated protein kinase 3 *(MAPK3), CCNB1, CDK1*, and cyclin *D1 (CCND1)* were significantly higher and *BCL-2* was significantly lower in calcium ionophore (CI)-treated human *in vitro *maturation-meiosis II (IVM-MII) oocytes (*P<0.05, **P<0.001).

In the present study, the transcript profiles of several oocyte maturation-related genes (*MAPK3, CCNB1, CDK1, and CCND1*) were evaluated by real-time RTPCR. The results showed that *MAPK3, CCNB1, CDK1*, and *CCND1 *mRNA expression levels compared with the housekeeping gene (*β-actin*) were up-regulated significantly in CI-treated oocytes (P<0.05; Fig.1). These findings led to the hypothesis that exposure of CI to human oocytes resulted in an apparent up-regulation in *MAPK3, CCNB1, CDK1*, and *CCND1* mRNA expressions.

A molecular mechanism that modulates human oocyte apoptosis might be induced by CI treatment. Therefore, we evaluated the *BCL-2, BAX*, and *Caspase-3 *relative expression levels by real-time RT-PCR. The results of real-time RT-PCR demonstrated that the expression of anti-apoptotic *BCL-2* was remarkably up-regulated after treatment with CI (P=0.001; Fig.1), whereas the expression of pro-apoptotic *BAX *did not change significantly (P=0.76). Thus, the *BAX/BCL-2* ratio decreased (13.60%). Also, real-time RT-PCR revealed that the expression level of Caspase-3 mRNA did not change significantly in human oocytes after exposure to CI (P=0.81; Fig.1).

### Effects of pre-*in vitro *maturation calcium ionophore treatment on glutathione and oxidative stress of in vitro-matured human oocytes

**Fig.2:**
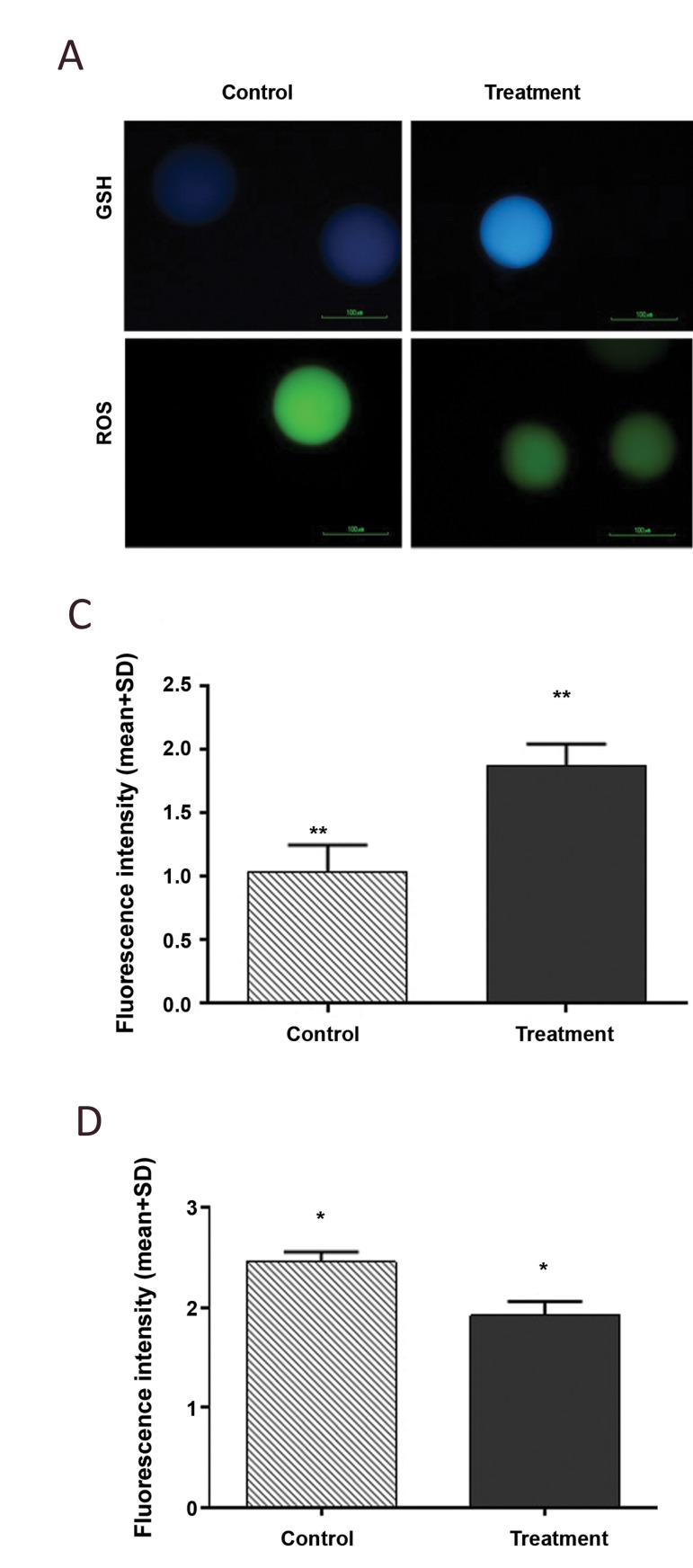
Glutathione (GSH) and reactive oxygen species (ROS) content in human *in vitro *maturation-meiosis II (IVM-MII) oocytes evaluated by fluorescent staining. **A.** Oocytes were stained with Cell Tracker Blue to assess the level of intracellular GSH and **B.** 2-7-dichlorodihydrofluorescein diacetate (H2DCFDA) to determine ROS (bar: 100 μm). **C.** GSH and **D. **ROS content in calcium ionophore (CI)-treated human oocyte and control groups. The data were analysed using the t test. As the graph depicts, CI‐treated oocyte cytoplasm had significantly higher GSH and lower ROS content (**P<0.01, *P<0.05).

The human oocyte GSH content was evaluated in the CI treatment and control groups. Analyses with ImageJ software indicated that CI treatment induced a statistically remarkable increase in oocyte intracellular GSH concentration (P=0.005, Fig .2A, B). A comparison of the intracellular ROS content of human oocytes (23 oocytes in each group) revealed significantly diminished ROS content in CI‐treated oocytes compared with the control group (P=0.04; Fig.2C, D).

**Fig.3:**
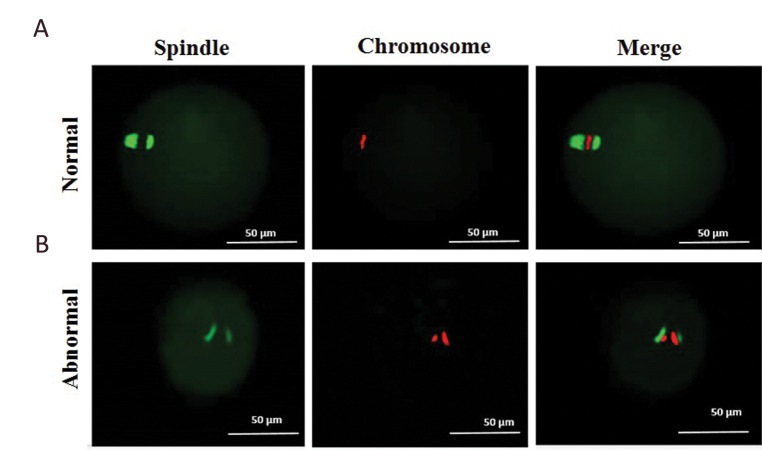
Meiotic spindle configuration and chromosome alignment in human *in vitro *maturation-meiosis II (IVM-MII) oocytes evaluated using immunocytochemistry. IVM-MII oocytes in calcium ionophore (CI)-treated human oocyte and control groups were fixed and stained for β-tubulin (green) and chromosomes (propidium iodide [PI], red), and analysed for meiotic spindle configuration and chromosome alignment. Meiotic spindles were classified as **A. **Normal with aligned chromosomes at the metaphase plate with barrel-shaped spindles and **B.** Abnormal with misaligned chromosomes in the metaphase plate with non-barrel-shaped spindles (bar: 50 μm).

### Effects of pre-*in vitro *maturation (pre-IVM) calcium ionophore (CI) treatment on chromosome alignment and meiotic spindle structure of in vitro-matured human oocytes

In order to find out whether the CI treatment could affect chromosome and spindle structure in human oocytes after IVM, we stained IVM-MII oocytes for β-tubulin to assess spindle configuration and PI to detect chromosomes. A total of 20 oocytes (10 oocytes in each group) were examined for meiotic spindle structure and chromosome alignment. Following the evaluation of β-tubulin positive spindles by fluorescent microscopy, one abnormal chromosome and spindle structure were observed in each group. There was no significant difference in normal spindle configuration and chromosome alignment rate (normal oocytes/ examined oocytes) between the CI‐treated group (90%) and control group (90%, Fig .3). This result showed that meiotic spindle bipolarity and chromosome alignment of human IVM-MII oocytes was not significantly affected by CI treatment.

## Discussion

Due to the absence of ovarian niches, human oocyte maturation following IVM is suboptimal. Some studies have reported morphological and structural differences after IVM of human oocytes in comparison with *in vivo* oocytes (2, 3).

Although in previous animal and human studies the influence of Ca^2+^ on oocyte maturation has been identified, its central role in human IVM as a mediator of MAPK, MPF, and apoptosis signalling cascade has not been proven. 

In this study, in order to demonstrate the effect of CI in oocyte maturation, we used CI before oocyte meiosis resumption during 24-28 hours of IVM. The results showed beneficial effects of CI on increasing nuclear maturation and anti-apoptotic gene expressions and cytoplasmic maturation. 

The effects of CI on human artificial oocyte activation have been shown before (20, 24). To our knowledge, this study is the first to identify the effects of CI on IVM of the human oocyte.

Promotive effects of CI on the IVM of human oocyte can be through several pathways.

The results of the present study demonstrated that CI up-regulates *MAPK, Cyclin B*, and *CDK1* gene expressions. These findings support the report of Liu et al., which stated that the cortical distribution of the calcium-sensing receptor regulated by gonadotropins in porcine oocytes improved oocyte IVM through the MAPK-dependent signalling cascade (25). The current study demonstrated that CI up-regulated *MAPK*, which then improved human oocyte maturation. This process might occur via the MAPK-related pathway. Protein kinase C (PKC) is the Ca^2+^ target downstream molecule (26). Cell cycle regulation by PKC cascades is involved in the activation of MAPK and MPF. CDK1 and cyclin B1 are PKC substrates. PKC inhibitor decreases MPF activity in the oocyte and PKC regulates MAPK signalling (6). It has been shown that MAPK is activated in cumulus cells by PKC activators (4). The present study findings contradict a previous observation by Ito et al. in which porcine oocytes were parthenogenetically activated by CI. They reported that *MAPK* activity decreased after pronucleus formation (27). It should be considered while we evaluated the MAPK levels in MII stage oocytes; the latter study reported the MAPK levels decreased after fertilization. Zhang et al. reported that MAPK levels increased during oocyte maturation until the MII stage, but the levels decreased after fertilization (28). 

The *CCNB1* expression level in the oocyte is a marker for cytoplasmic maturation (25). Liang et al. showed that stored mRNA of *CCNB1 *in the cytoplasm of the oocyte could influence MAPK and the MPF pathway (29). These results indicated that CI could increase cytoplasmic maturation in IVM-MII oocytes by enhancement of MAPK activity. The finding of the present study supported their views. 

The relative expression level of *CCND1*, a cell cycle regulator gene, is a proliferative marker. Increasing expression of *CCND*1 has been reported during meiosis progression in mouse oocytes (31). In mammalian oocytes, *CCND1* was expressed both in the oocyte and granulosa cells during follicular growth (30) and has a crucial role in follicles and granulosa cell proliferation, survival, and early embryonic transition (30). Gatius et al. (32) showed that MAPK signalling promotes cell proliferation by activation of CCND1. Up-regulation of *MAPK* in the present study might be the result of the activation of *CCND1*.

In general, oxidative stress induced by overloading of Ca^2+^ is an apoptotic signal that can increase BAX/BCL-2 and increase apoptosis in the oocyte (14). The findings of the present study show that CI could upregulate antiapoptotic BCL-2. It does not up-regulate pro-apoptotic *BAX *and effector *Caspase-3* gene expression in IVM-MII human oocytes. In agreement with our findings, several studies have shown that decreased levels of MAPK and MPF in oocytes also lead to increased BCL-2 protein degradation and activation of the apoptotic pathway in mice (15), rat (13), and canine (33) oocytes. Also, it has been reported that inhibition of CDK1 activity by reducing the MPF heterodimer prevents meiotic cell cycle progression and induces apoptosis (13, 15). Decreased CDK1 phosphorylation as well as increased degradation of cyclin B1 lead to MPF instability and result in fas ligand-induced apoptosis in oocytes (14). Thus, the increased expression levels of MAPK and MPF genes in our study might be responsible for an increased survivalpromoting signalling in IVM-MII oocytes. Tripathi and Chaube added different concentrations of CI (0.5, 1, 2, 3, 4 μM) to rat MII oocyte culture medium for 3 hours and showed that high concentrations (3 and 4 μM) of CI led to increased ROS production and apoptosis in oocytes (34). Moreover, Chaube et al. reported that the addition of CI (1.6 μM) to the culture medium of rat MII stage oocytes for 3 hours induced hydrogen peroxide formation and apoptosis in these oocytes (35). In both of these studies, the oocyte developmental stage, CI concentrations, and exposure duration were not similar to our work.

In the current study, we observed higher GSH and lowered ROS content in CI‐treated oocyte cytoplasm. Intracellular GSH concentration is an oocyte cytoplasmic maturation marker. Increasing GSH synthesis in oocytes starts from meiosis resumption in the GVBD stage and reaches its highest concentration at the MII stage (19). GSH regulates many processes in the oocyte, including modulating the intracellular redox balance, defending oocytes from ROS damage, influencing sperm nuclear decondensation, and male pronucleus formation, DNA synthesis, and amino acid and protein transport (11). BCL-2 prevents the intrinsic apoptotic pathway in mitochondria. Besides its anti-apoptotic function, BCL2 has an antioxidant-like property that has been related to the regulation of the intracellular concentration of *GSH*. Previous studies have reported that increased BCL2 expression causes an increase in intracellular GSH content by enhanced GSH synthesis and reduced cellular GSH efflux (36). In our research, overexpression of BCL2 induced by CI treatment might be the reason for the increase in GSH content and, subsequently, reduced ROS status in oocytes after IVM.

In the present study, we showed that CI did not disturb the meiotic spindle structure and chromosome alignment. Abnormal spindle assembly and chromosome segregation cause aneuploidy in oocytes, which leads to the embryo development arrest and spontaneous abortion (37). Our finding might be due to the MAPK and MPF pathway that has a significant role in the remodeling of actin filaments and microtubule organization (4, 25). In agreement with our findings, Luo et al. (38) showed that inhibition of the activation of MAPK during porcine oocyte maturation resulted in prevention spindle microtubules assembly and first PB extrusion. Choi et al. (39) reported that increased oxidative stress and a decreased intracellular concentration of GSH led to the spindle structure defect in IVM mouse oocytes. Nevertheless, the normal spindle morphology was reported in IVM-MII macaque oocytes, which GSH ethyl ester was added to the IVM culture medium (10). Considering the protective effect of GSH on the meiotic spindle structure and cytoplasmic microtubules, CI might prevent the meiotic spindle disruption and chromosome misalignment in IVM-MII human oocytes through increased levels of the intracellular GSH level.

We did not find any effect of CI on the first PB extrusion in human oocytes. In contrast to our finding, Makki et al. (40) reported that addition of 15 μg/ml selenium, 10 μg/ ml calcium, and 5 μg/ml CI to the IVM medium for 24 hours improved IVM and fertilization of oocytes, and the embryo cleavage rate. The differences between the findings of this study and our work might be due to the various times of exposure and compounds which were added to the culture medium.

In the current study, we showed that CI could improve oocyte cytoplasmic and nuclear maturation during IVM of human oocytes, but it could not alter the extrusion of the first PB of the oocytes. It should be mentioned that we evaluated the expression of genes related to maturation in oocytes at the RNA level, whereas the first PB extrusion was regulated when these RNAs were translated into protein. Hence, it seemed that the prolonged *in vitro *culture of the oocyte might lead to the conversion of maturation related RNA genes to proteins and improve the first PB extrusion of the oocytes. Therefore, further clarification of the impact of the CI on maturation related proteins is required.

## Conclusion

The finding of the current study seems to supports the beneficial effect of CI on the developmental competence of human oocytes, including nuclear and cytoplasmic maturation, and apoptosis of human oocytes. We suggest that the CI may optimize the human IVM procedure in the ART clinic.
